# The Fab Fragment of a Human Anti-Siglec-9 Monoclonal Antibody Suppresses LPS-Induced Inflammatory Responses in Human Macrophages

**DOI:** 10.3389/fimmu.2016.00649

**Published:** 2016-12-26

**Authors:** Sasa Chu, Xuhui Zhu, Na You, Wei Zhang, Feng Zheng, Binggang Cai, Tingting Zhou, Yiwen Wang, Qiannan Sun, Zhiguo Yang, Xin Zhang, Changjun Wang, Shinan Nie, Jin Zhu, Maorong Wang

**Affiliations:** ^1^Department of Infectious Disease, Anhui Medical University Affiliated with Bayi Clinical College, Hefei, China; ^2^Institute of Liver Disease, Nanjing Jingdu Hospital, Nanjing, China; ^3^Huadong Medical Institute of Biotechniques, Nanjing, China; ^4^Department of Emergency Medicine, Jinling Hospital, Medical School of Nanjing University, Nanjing, China; ^5^Department of Traditional Chinese Pharmacology, Chinese Pharmaceutical University, Nanjing, China; ^6^Department of Pathology, Key Laboratory of Antibody Technique of the Ministry of Health, NJMU, Nanjing, China

**Keywords:** Siglec-9, human anti-Siglec-9 antibody, Fab fragment, LPS, TLR4, sepsis, human macrophages

## Abstract

Sepsis is a major cause of death for hospitalized patients and is characterized by massive overreaction of immune responses to invading pathogens which is mediated by cytokines. For decades, there has been no effective treatment for sepsis. Sialic acid-binding, Ig-like lectin-9 (Siglec-9), is an immunomodulatory receptor expressed primarily on hematopoietic cells which is involved in various aspects of inflammatory responses and is a potential target for treatment of sepsis. The aim of the present study was to develop a human anti-Siglec-9 Fab fragment, which was named hS9-Fab03 and investigate its immune activity in human macrophages. We began by constructing the hS9-Fab03 prokaryotic expression vector from human antibody library and phage display. Then, we utilized a multitude of assays, including SDS-PAGE, Western blotting, ELISA, affinity, and kinetics assay to evaluate the binding affinity and specificity of hS9-Fab03. Results demonstrated that hS9-Fab03 specifically bind to Siglec-9 antigen with high affinity, and pretreatment with hS9-Fab03 could attenuate lipopolysaccharide (LPS)-induced TNF-α, IL-6, IL-1β, IL-8, and IFN-β production in human PBMC-derived macrophages, but slightly increased IL-10 production in an early time point. We also observed similar results in human THP-1-differentiated macrophages. Collectively, we prepared the hS9-Fab03 with efficient activity for blocking LPS-induced pro-inflammatory cytokines production in human macrophages. These results indicated that ligation of Siglec-9 with hS9-Fab03 might be a novel anti-inflammatory therapeutic strategy for sepsis.

## Introduction

Sepsis is a leading cause of mortality in intensive care units; recent statistics have indicated the occurrence about 19 million cases worldwide per year (1). Current treatments for sepsis are typically supportive and often ineffective, despite the fact that the number of deaths per year is around eight million (2). Moreover, more than 30% of survivors suffer from long-term functional disabilities and persistent critical illness (3). Lethality caused by sepsis arises from a massive hyperinflammatory immune response to pathogens, such as Gram-negative organisms, Gram-positive organisms, and fungi (4). The uncontrolled pro-inflammatory responses that lead to organ dysfunction in sepsis are primarily initiated by the toll-like receptors (TLRs), which recognize pathogen-associated molecular patterns (PAMPs), such as bacterial lipoproteins, lipopolysaccharide (LPS), and non-methylated CpG DNA (5). TLR4 activation results in the synthesis and release of pro-inflammatory cytokines, such as TNF-α, IL-6, IL-8, and IFN-β, which act locally but are released systemically, initiating the cytokines storm that damage vital tissues (6, 7). Our recent studies indicated that anti-TLR4 Fab fragment could reduce the inflammatory responses by inhibiting LPS-induced TLR4 signaling pathway in mouse primary macrophages and human THP-1-differentiated macrophages (8, 9). Therefore, it is intriguing that macrophages and inflammatory cytokines could be potential therapeutic targets in patients with sepsis.

Sialic acid-binding immunoglobulin-type lectins (Siglecs) are a family of sialic acid-binding immunoglobulin-like lectins that are differentially presented on the surface of hematopoietic cells, which exert immunomodulatory functions *via* glycans or glycoproteins recognition during immune responses (10, 11). Siglecs can be categorized into two groups. CD169, CD22, MAG, and Siglec-15 are conserved across mammals. In comparison, the CD33-related Siglecs are variable across mammals (12). It has been suggested that CD33-related Siglecs may serve as a negative regulator for immunocytes behavior, such as inhibition of cellular activation, induction of apoptosis, and suppression of pro-inflammatory cytokines production (13). All of CD33-related Siglecs may transduct through their immunoreceptor tyrosine-based inhibitory motifs (ITIMs) located in the cytoplasmic region (except for Siglec-14), which are associated with SHP-1 and/or SHP-2 (14, 15). Siglec-9, as a member of the CD33-related Siglecs, is predominantly presented on neutrophils, monocytes, macrophages, and dendritic cells (DCs), whose mouse ortholog Siglec-E are expressed on neutrophils, monocytes, and conventional dendritic cells (16, 17). Siglec-9 has a characteristic N-terminal, Ig-like, V-type domain which could mediate its binding to sialic acid moiety of glycans and glycoproteins, a single transmembrane region, and a cytoplasmic tail that contain an ITIM and SLAM-like motif (18, 19). It is well established that ligation of the Siglec-9 induces phosphorylation of the tyrosine within the ITIM and recruit tyrosine phosphatase SHP-1 and SHP-2, then exerts its inhibition during innate and acquired immunity (20).

The cross talks between Siglecs family and TLRs are under intense investigation. Recently, Siglecs expressed on neutrophils, macrophages, and DCs could regulate TLRs-induced cytokines production through small RNA interference or *in vitro* ligation with Siglecs-specific antibodies. Results showed that Siglec-G could not regulate responses to microbial products directly, but instead it might interact with the receptor CD24 in *cis* to inhibit DC-initiated inflammatory reactions (21). Chen et al. showed that Siglec-G expression could be upregulated on macrophages after infection by vesicular stomatitis virus (VSV) or Sendai virus, which lead to the degradation of retinoic acid-inducible gene I and inhibition of the IFN-β production (22). Furthermore, recent results suggest that Siglec-9 inhibits the production of TNF-α while promotes the secretion of the IL-10 upon stimulation with LPS in macrophages, but the precise mechanism of Siglec-9-influenced LPS signaling pathway is still unknown (23).

Thus, we prepared the Fab fragments of human anti-Siglec-9 monoclonal antibody (hS9-Fab03) from human antibody library and phage display and examined whether treatment of hS9-Fab03 could regulate immune responses upon stimulation by LPS in human macrophages. In this study, we report that hS9-Fab03 not only attenuates LPS-induced TNF-α, IL-6, IL-1β, IL-8, and IFN-β production in human PBMC-derived macrophages but also slightly increases IL-10 production in an early time point.

## Materials and Methods

### Cells and Reagents

The THP-1 cells were acquired from the cell bank of Shanghai Institute of Biochemistry and Biology (Chinese Academy of Sciences, Shanghai, China). RPMI-1640 medium and fetal bovine serum (FBS) were obtained from Gibco (Carlsbad, CA, USA). LPS (O111:B4), PMA, Ficoll-Paque Plus, and commercial anti-Siglec-9 antibody were obtained from Sigma-Aldrich (St. Louis, MO, USA), Abs specific to GAPDH, total p38, phosphorylated JNK1/2, p38, p65, and IRF-3 were purchased from Cell Signaling Technology (Danvers, MA, USA). His-trap Lambda Fab Select column was obtained from GE Healthcare (Piscataway, NJ, USA). Anthrax chimeric Fab antibody was prepared in our lab.

### Cell Culture and Differentiation

THP-1 cells were cultured in RPMI-1640 supplied with 10% FBS, 1% penicillin, and streptomycin in a 5%-CO_2_ humidified incubator at 37°C. The THP-1 cells were stimulated with 10 nM PMA for 48 h, then THP-1-derived macrophages were differentiated. The PBMC-derived macrophages were cultured and differentiated as previously reported (24). Briefly, human PBMCs were separated by centrifugation on Ficoll-Paque Plus and purified by CD14-positive cells isolation kit (Miltenyi Biotec, CA, USA). The purified cells were differentiated in complete RPMI-1640 supplied with M-CSF (10 ng/ml) (BD Biosciences, CA, USA) for 6 days. Donor blood samples were randomly collected in the Jiangsu Province Blood Center. The study was approved by Ethical Committee of Anhui Medical University Affiliated with Bayi Clinical College and all participants signed an informed consent form when they filled the questionnaire.

### Phage Library and Helper Phage

A human naive Fab phage library for Siglec-9 selection was generated as previously described (25). Before the first round panning, the phage library was titrated and 1 × 10^13^ phage clones were collected for panning.

### Phage ELISA

Single phage clone from the *E. coli* XL1-Blue infected by the seventh round of eluted phage was randomly picked up and grown in 1 ml super broth (SB) medium containing 100 µg/ml ampicillin and 1% glucose. VCSM13 helper phage (1 × 10^9^) was added to each vial. Then, 50 µl of supernatant from each vial was added to each well of 96-well plate, which was pre-coated with 100 ng extracellular domain of Siglec-9 antigen. After incubation for 2 hours and washing for three times, 50 µl of horseradish-peroxidase (HRP)-conjugated anti-M13 antibody solution was added to each well. Finally, the highest absorbance of the positive clones was selected for further evaluation.

### Construction of the hS9-Fab03 Expression Vector

Total RNA was extracted from positive cells by the TRIzol reagent (Invitrogen, CA, USA) and converted to complementary DNA (cDNA) by the Prime Script RT Master Mix kit (TaKaRa, Shiga, Japan) according to the manufacturer’s protocol. The variable regions of the heavy (V_H_) chain and the light (V_L_) chain were amplified with primers shown in Table S1 in Supplementary Material. The conserved regions of the heavy (C_H_1) and light (C_L_) chains were provided by the Barbas laboratory (Scripps Research Institute, USA). The V_H_ and V_L_ were amplified and cloned into pMD-18T vector, respectively. The heavy chain Fd and the light chain L were amplified with HF1, HR2, and LL1, LR2, respectively. The Fd and L were cloned into pETDuet-1 at *Nco*I/*Hin*dIII and *Eco*RV/*Xho*I, respectively.

### Western Blotting

The expression of hS9-Fab03 in *E. coli* BL21 was detected by Western blotting as previously described (26). The concentrations of proteins were determined by bicinchoninic acid protein assay kit (Thermo, Waltham, MA, USA). The proteins from bacteria lysate were dissociated by 12% SDS-PAGE and then transferred onto nitrocellulose membrane (Bio-Rad, CA, USA). The membrane was blocked by 5% non-fat milk in TBST for 1 h at room temperature, which was then incubated with HRP-conjugated goat anti-human antibody (Fab specific) (Santa Cruz Biotechnology, CA, USA). The THP-1-derived macrophages (1 × 10^6^ cells/well) were pretreated with the hS9-Fab03 (5 µg/ml) for 2 h and stimulated with LPS (1 µg/ml) for 0, 15, 30, and 60 min. The phosphorylation level of NF-κB/MAPKs/IRF-3 signaling pathway were detected and analyzed.

### Fluorescence-Activated Cell Sorting (FACS)

The THP-1-derived macrophages were treated with the hS9-Fab03 for 2 h; the cells were then incubated with goat anti-human IgG (Fab specific)-FITC antibody (Sigma-Aldrich, MO, USA) at 37°C for 1 h. The cells were analyzed by LSRII flow cytometer (BD Biosciences, CA, USA).

### ELISA

TNF-α, IL-1β, IL-6, IL-10, and IL-8 in supernatants were determined with ELISA kits (R&D Systems, MN, USA).

### Surface Plasmon Resonance (SPR) Analysis of the hS9-Fab03

We optimized the coupling condition based on the isoelectric point of the Siglec-9 protein, and sodium acetate was chosen as the dilution buffer for Biacore X100 SPR system (GE, Sweden). The Siglec-9 protein was diluted to 30 µg/ml and coupled to the CM5 chip. The coupling level was set at 1,500 RU, the injection time was set to 180 s, the dissociation time was set to 15 min, and 50 mM Gly-HCl (pH = 1.7) was used as the regeneration buffer. Finally, the Siglec-9 protein was treated with serial concentrations of hS9-Fab03 to detected the antibodies’ binding affinity.

### Real Time Q-PCR

The total RNA was extracted using RNAfast200 (Feijie, Shanghai, China) following the manufacturer’s instructions. A quantity of 0.5 µg total RNA was used in a 10-µl reverse-transcription reaction using the PrimeScript RT Master Mix kit, then the cDNA was diluted into 40 µl as template for real time Q-PCR. Q-PCR analysis was performed using LightCycler (Roche Diagnostic, IN, USA). The primers used for human GAPDH, TNF-α, IL-6, IL-1β, IL-8, IL-10, and IFN-β were as described previously (27). Data were normalized by the level of GAPDH expression in each sample.

### Statistical Analysis

The significance of differences was analyzed by Student’s *t*-test and Mean ± SD was calculated, with a value of *p* < 0.05 considered to be statistically significant.

## Results

### Preparation of Human Anti-Siglec-9 Fab Fragments

**Figure 1:**
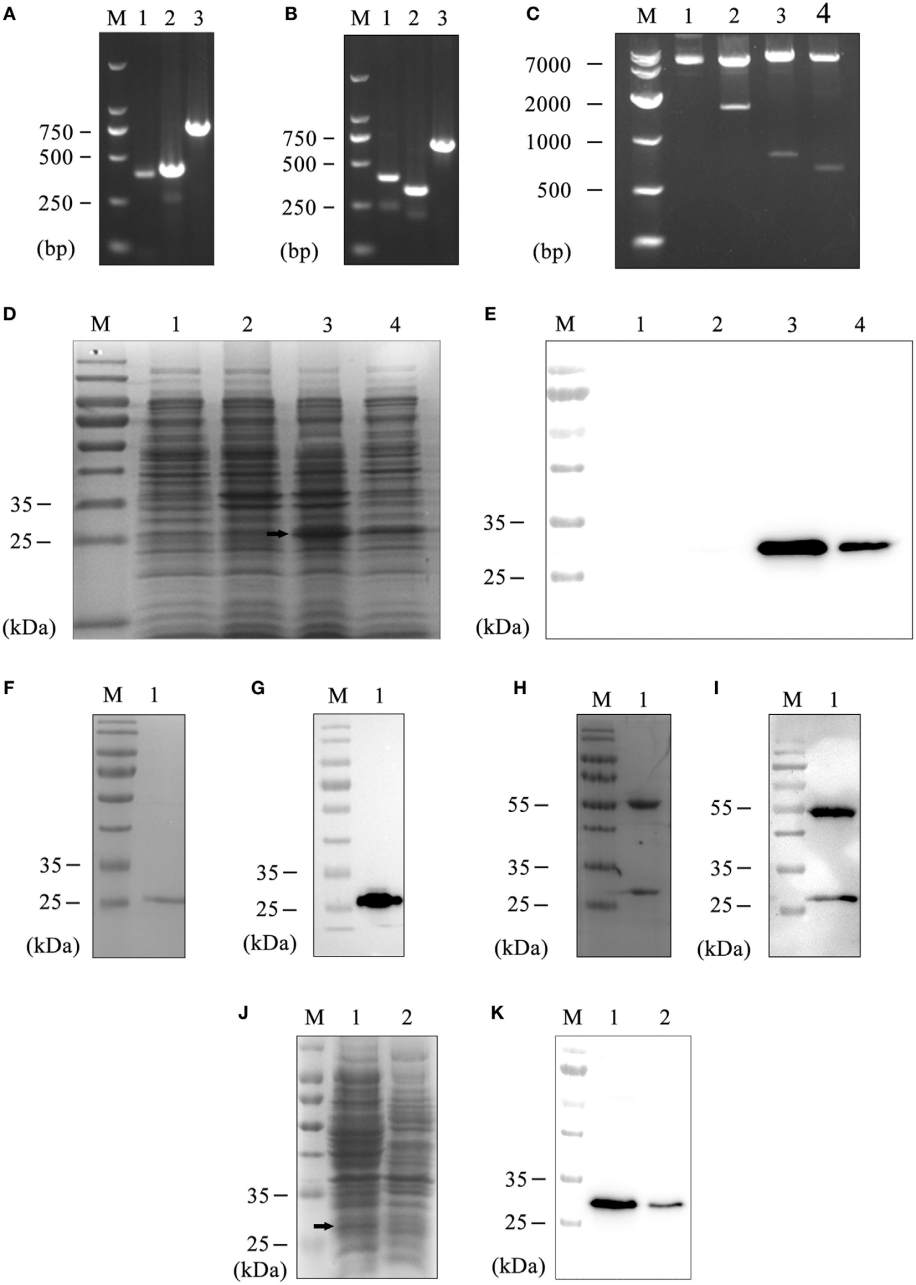
**Preparation of the hS9-Fab03**. **(A)** The heavy chain PCR products of positive clone. Lane 1, V_H_; Lane 2, C_H_; Lane 3, V_H_ combined with C_H;_ Lane M, DNA maker. **(B)** The light chain PCR products of positive clone. Lane 1, V_L_; Lane 2, C_L;_ Lane 3, V_L_ combined with C_L_; Lane M, DNA maker. **(C)** The construction of pETDuet-hSiglec-9 Fab03. Lane 1, the plasmid without restriction endonuclease digestion; Lane 2, the plasmid was double digested with *Nco*I and *Xho*I; Lane 3, *Nco*I and *Hin*dIII were used for double digesting the plasmid; Lane 4, *Eco*RV and *Xho*I were used for double digesting the plasmid; Lane M, DNA marker. Coomassie blue staining **(D)** and Western blotting **(E)** detected the expression of the recombinant vector. Lane 1, whole lysate of untransfected *E. coli* BL21, as a negative control; Lane 2, whole lysate of pETDuet-hSiglec-9 Fab03-transfected *E. coli*; Lane 3, supernatant of sonicated lysate of pETDuet-hSiglec-9 Fab03-transfected *E. coli*; Lane 4, sediment of sonicated lysate of pETDuet-hSiglec-9 Fab03-transfected *E. coli*; Lane M, protein marker. All strains were induced by IPTG overnight. Coomassie blue staining **(F)** and Western blotting **(G)** detected the purified Fab fragments. Lane 1, supernatant of sonicated lysate of pETDuet-hSiglec-9 Fab03-transfected *E. coli* induced by IPTG overnight; lane M, protein marker. **(H)** Coomassie blue staining showed that the hS9-Fab03 was expressed, and the heavy chain Fd and light chain L were linked together. **(I)** horseradish-peroxidase-conjugated goat anti-human antibody (Fab specific) was used to detect the heavy chain Fd, and light chain L of the hS9-Fab03 was expressed and separated in Western blotting. Coomassie blue staining **(J)** and Western blotting **(K)** detected the efficiency of hS9-Fab03. Lane 1, supernatant of sonicated lysate of the pETDuet-hSiglec-9 Fab03-transfected *E. coli* was purified by the His-trap Lambda Fab Select column; lane 2, whole lysate of pETDuet-hSiglec-9 Fab03-transfected *E. coli* induced by IPTG overnight; lane M, protein marker.

Generally, with seven rounds of affinity panning, 50 single phages were selected and analyzed by phage ELISA. While the positive and negative ratio was exceeded 4, the phage was considered positive. Ten positive phages were possessed, amplified, and confirmed by sequencing. After examining the sequence in VBASE2 database, the light chain was classified as lambda chain. Consequently, we amplified the V_L_ and C_L_ of selected positive clones with universe primers, which were designed by our lab as previously reported (28). The length of V_H_ and C_H_1 were close to 400 bp (Figure 1A). Similarly, the length of V_L_ and C_L_ were approximately 400 and 350 bp, respectively (Figure 1B). Then, the heavy chain Fd and light chain L were spliced by overlap extension-polymerase chain reaction (29), which were approaching to 800 and 750 bp, respectively. Finally, the Fd and L fragments were inserted into the prokaryotic expression plasmid pETDuet-1 sequentially affirmed by digestion and sequencing (Figure 1C). Therefore, the prokaryotic expression vector of human anti-Siglec-9 Fab fragments (pETDuet-hSiglec-9 Fab03) had been constructed successfully.

The vector of pETDuet-hSiglec-9 Fab03 was transformed into *E. coli* BL21 and induced with 1 mmol/l IPTG overnight at 16 or 37°C, the recombinant *E. coli* was preferred to express more Fab fragments (data not shown). Then the lysate of the recombinant *E. coli* was examined by SDS-PAGE gel with Coomassie brilliant blue staining, the bands of Fd and L were overlapped due to their similar DNA length (Figure 1D). To determine whether the Fab fragments were expressed in the inclusion body, western blotting was utilized. We observed that the recombinant proteins were primarily secreted in the component of ultrasonic supernatant but not that of sediment (Figure 1E). After purification, the Fab antibodies were presented at 27 kDa (Figures 1F,G). Compared with SDS-PAGE electrophoresis, Native-PAGE could reduce the possibility of protein denaturalization. As shown in Figures 1H,I, results showed that two obvious bands were at 27 and 55 kDa, respectively.

Considered to light chain of the recombinant protein was lambda chain; the purification of the human anti-Siglec-9 Fab fragments (hS9-Fab03) was achieved by His-trap Lambda Fab Select column. The purification efficiency of target proteins was above 95% with the concentration of hS9-Fab03 up to 1.0 mg/ml (Figures 1J,K). Collectively, the hS9-Fab03 was successfully expressed at high concentration to explore its affinity and immune activity.

### The hS9-Fab03 Specifically Binds Siglec-9

**Figure 2:**
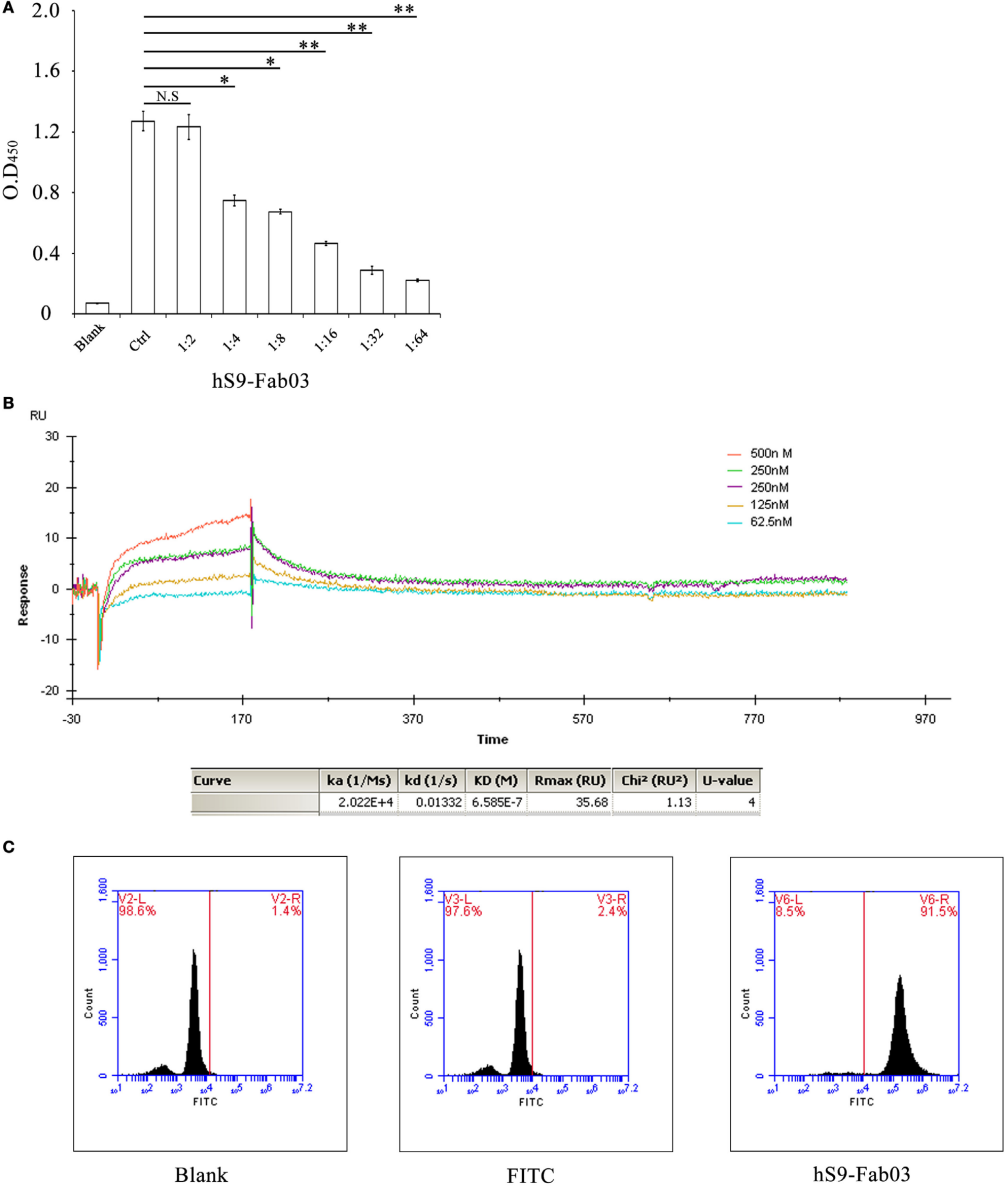
**The hS9-Fab03 specifically binds the Siglec-9 antigen**. **(A)** 96-well plates were pre-coated with recombinant human Siglec-9, 10 µg/ml. Serial concentrations of the hS9-Fab03 were used as the primary antibody. Horseradish-peroxidase-conjugated goat anti-human antibody (Fab specific) was used as the secondary antibody. Commercial anti-Siglec-9 antibodies were used as positive control (Ctrl). The absorbance was read at 450 nm after color development. **(B)** The hS9-Fab03 was diluted with buffer solution to 30 µg/ml and then treated with a running buffer containing different concentrations of hS9-Fab03. Results were analyzed using the Biacore X100 software. **(C)** PMA-stimulated THP-1-differentiated macrophages were treated or untreated with hS9-Fab03 at 4°C for 1 h, Goat anti-human IgG (Fab specific)-FITC antibody was incubated with macrophages for 1 h in the dark at 37°C. The cells were determined by fluorescence-activated cell sorting. Experiments were repeated in triplicate. Data are shown as mean ± SD (*n* = 3, N.S., not significant, *p < 0.05, **p < 0.01, ***p < 0.001 compared to control).

To examine whether the hS9-Fab03 could specifically bind to Siglec-9 antigen, the ELISA assay was carried out with different concentration of hS9-Fab03, and the commercial anti-Siglec-9 antibody was used as positive control. Next, the concentration of hS9-Fab03 was diluted from 0.5 to 0.07 mg/ml. The absorbance value at 450 nm was from 1.174 to 0.216 (Figure 2A). Finally, the ELISA results showed that the hS9-Fab03 could particularly bind to Siglec-9 antigen in a dose-dependent manner, which was as good as the commercial antibody.

Aiming to characterize the binding capacity of hS9-Fab03, we further calculated the affinity constant using the following formula: affinity constant (KD) = dissociation constant (Kd) ÷ binding constant (Ka) (30). Results from the Biacore X100 SPR analysis demonstrated that the affinity constant of hS9-Fab03 was 6.58 × 10^−7^ (Figure 2B). Taken together, these results indicated that hS9-Fab03 could effectively bind to Siglec-9 protein.

The binding ability of hS9-Fab03 was further analyzed with Flow cytometry in Siglec-9-positive THP-1-derived macrophages. Our results revealed that the population of hS9-Fab03-treated THP-1 cells was divided from unbinding cells, whereas no apparent difference was detected in blank and FITC antibody-treated cells (Figure 2C). This implied that hS9-Fab03 could effectively bind to Siglec-9 expressed on the cells’ surface.

### The hS9-Fab03 Inhibits LPS-Induced Inflammatory Cytokines Production *In Vitro*

Human macrophages have been widely used for studying TLRs signaling (31). To investigate the role of Siglec-9 during LPS-initiated inflammatory responses, we determined the efficient pre-incubated time and optimized concentrations of hS9-Fab03 and LPS. When the concentration of hS9-Fab03 range from 0.1 to 5 µg/ml, the inhibition efficiency of IFN-β and TNF-α mRNA levels were increased in a concentration-dependent manner (Figures S1A,B in Supplementary Material). Then, when the concentration of hS9-Fab03 was at 5 µg/ml, the pre-incubated time of hS9-Fab03 was ranging from 30 min to 4 h, the inhibition efficiency of IFN-β and TNF-α mRNA levels was better at 2 h (Figures S1C,D in Supplementary Material). Furthermore, treatment with 5 µg/ml of hS9-Fab03 reduced LPS-initiated inflammatory responses by approximately 85% of IFN-β expression and by approximately 70% of TNF-α expression than without antibody treatment (Figures S1E,F in Supplementary Material). Finally, we conducted the following experiments with pre-incubation time at 2 h and the concentration of hS9-Fab03 and LPS at 5 and 1 µg/ml, respectively.

**Figure 3:**
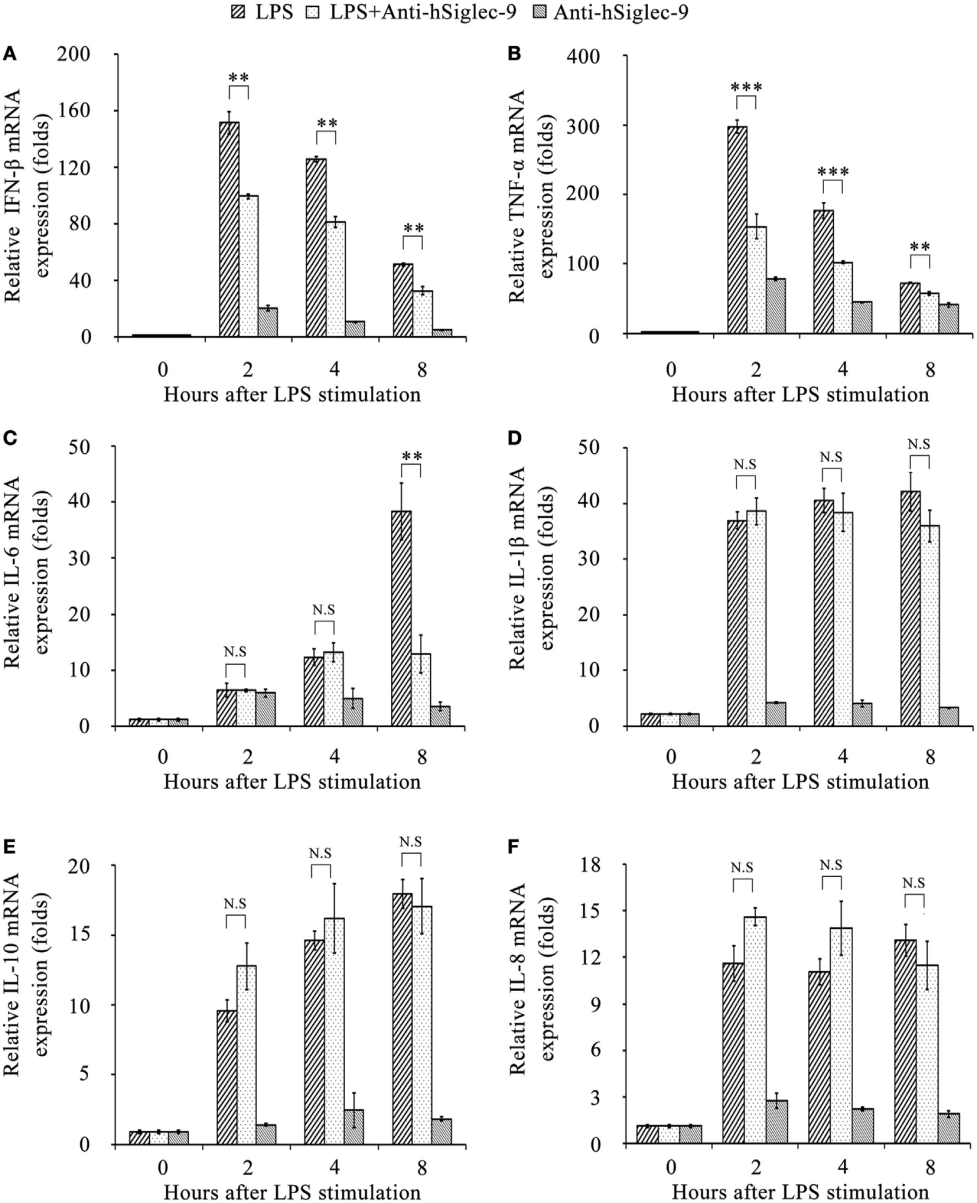
**The hS9-Fab03 inhibits lipopolysaccharide (LPS)-induced inflammatory cytokines production in THP-1-derived macrophages**. PMA-stimulated THP-1-differentiated macrophages in 24 wells were pre-incubated with 5 µg/ml hS9-Fab03 for 2 h, then stimulated with 1 µg/ml LPS. Anthrax chimeric Fab antibody was used as the negative control. The mRNA expression levels of IFN-β **(A)**, TNF-α **(B)**, IL-6 **(C)**, IL-1β **(D)**, IL-10 **(E)**, and IL-8 **(F)** were detected by Q-PCR. Experiments were performed in triplicate. Data are shown as mean ± SD (*n* = 3, N.S., not significant, *p < 0.05, **p < 0.01, ***p < 0.001 compared to negative control).

**Figure 4:**
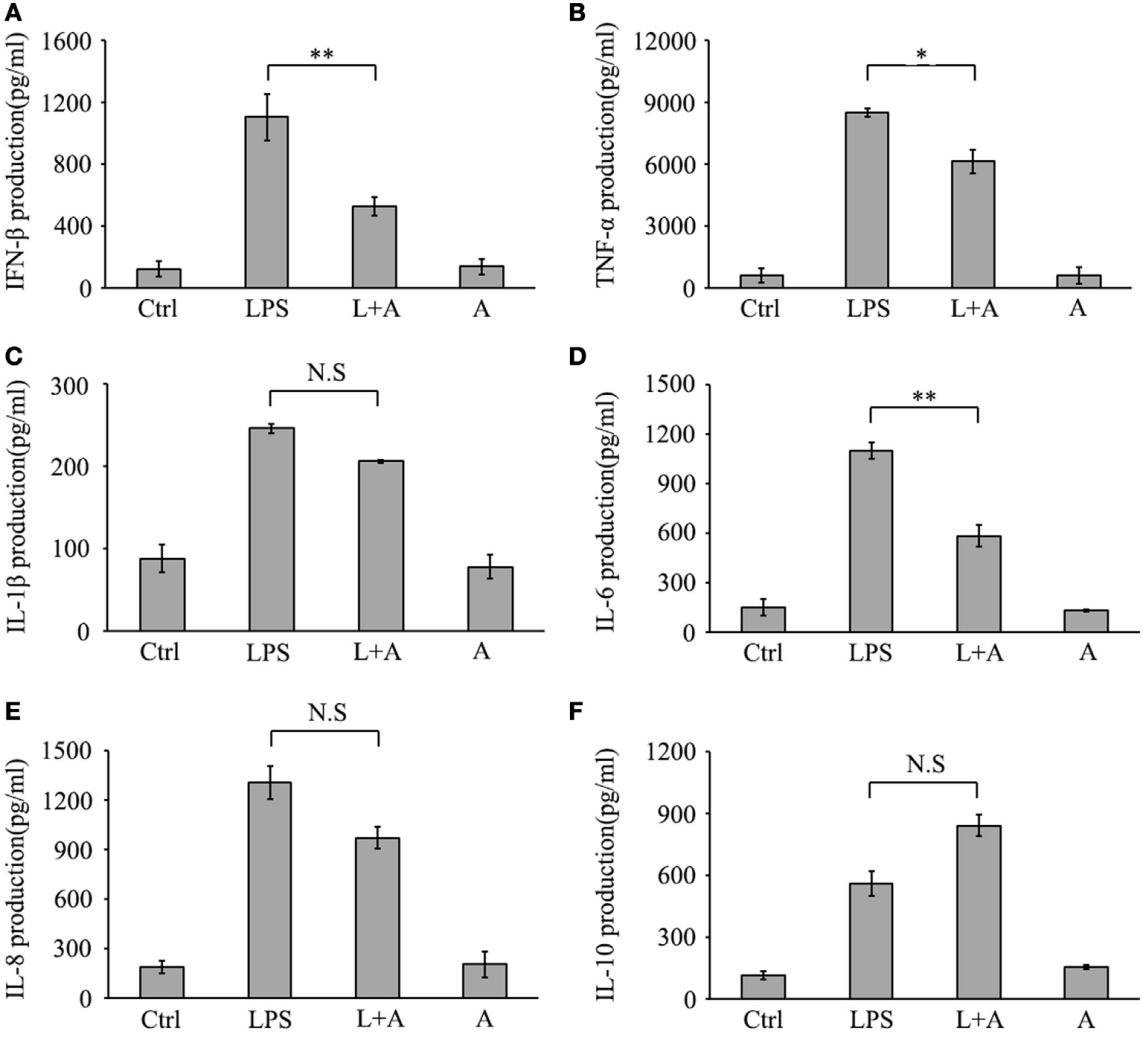
**Inhibitory effect of hS9-Fab03 on cytokine expression in cell culture supernatant of lipopolysaccharide (LPS)-stimulated THP-1-differentiated macrophages**. THP-1-differentiated macrophages were pre-incubated with 5 µg/ml hS9-Fab03 for 2 h, then stimulated with 1 µg/ml LPS. Anthrax chimeric Fab antibody was used as the negative control. The production of IFN-β **(A)**, TNF-α **(B)**, IL-1β **(C)**, IL-6 **(D)**, IL-8 **(E)**, and IL-10 **(F)** in human THP-1-differentiated macrophage culture supernatant was determined by ELISA analysis. Data are shown as mean ± SD (*n* = 3, N.S., not significant, *p < 0.05, **p < 0.01, ***p < 0.001 compared to negative control).

**Figure 5:**
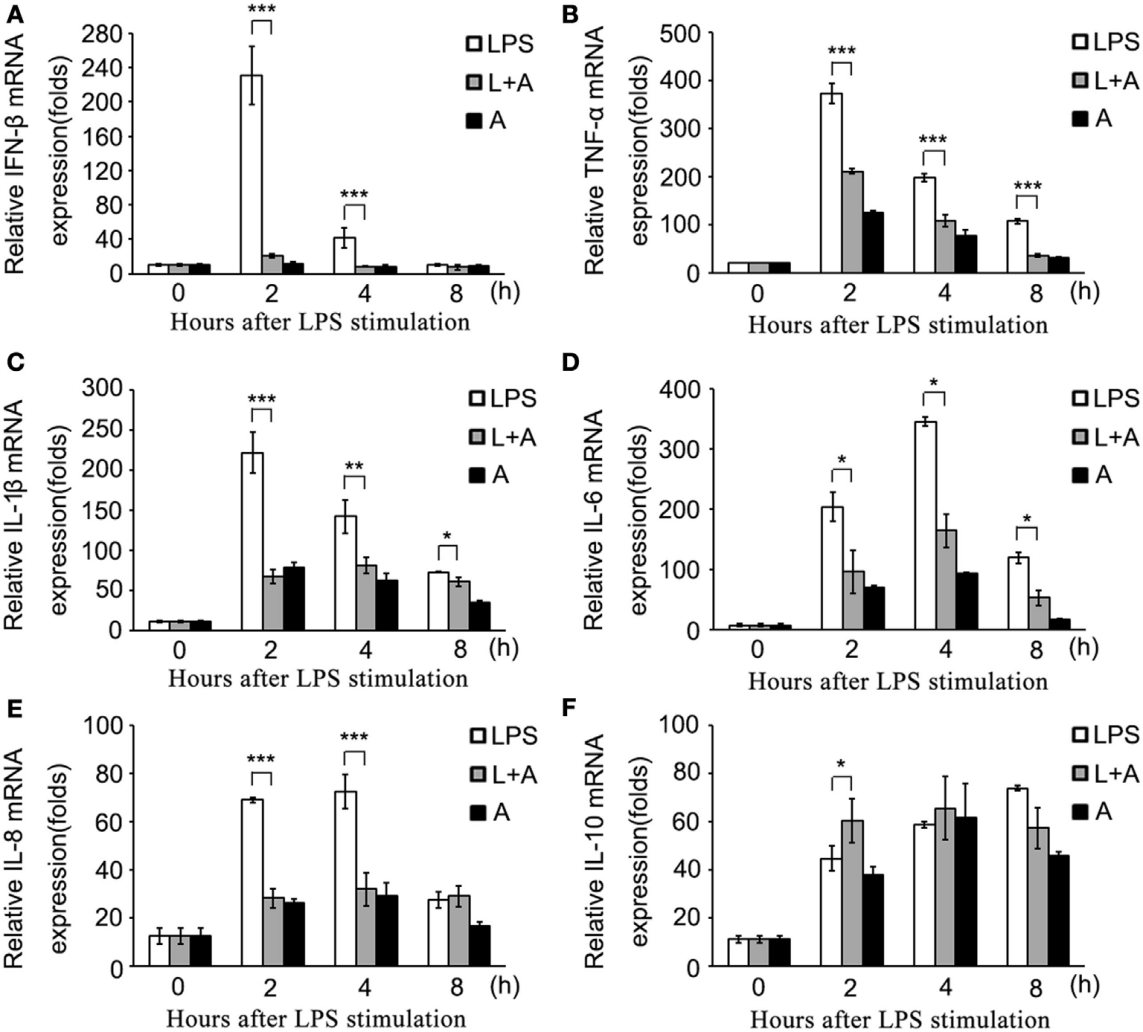
**The hS9-Fab03 inhibits lipopolysaccharide (LPS)-induced inflammatory cytokines production in human PBMC-derived macrophages**. Human PBMC-differentiated macrophages in 24 wells were pre-incubated with 5 µg/ml hS9-Fab03 for 2 h, then stimulated with 1 µg/ml LPS. Anthrax chimeric Fab antibody was used as the negative control. The mRNA expression levels of IFN-β **(A)**, TNF-α **(B)**, IL-1β **(C)**, IL-6 **(D)**, IL-8 **(E)**, and IL-10 **(F)** were detected by Q-PCR. Experiments were performed in triplicate. Data are shown as mean ± SD (*n* = 3, N.S., not significant, *p < 0.05, **p < 0.01, ***p < 0.001 compared to negative control).

**Figure 6:**
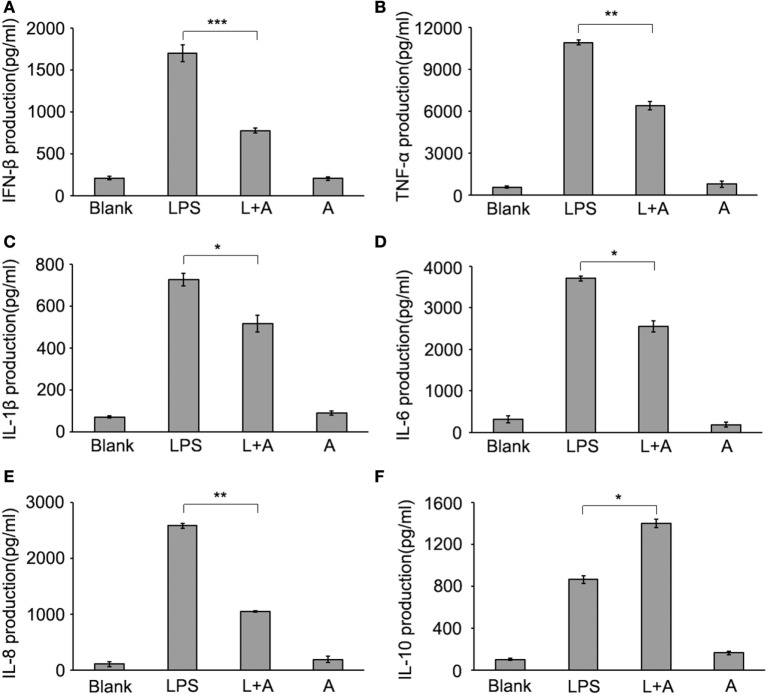
**Inhibitory effect of hS9-Fab03 on cytokine expression in cell culture supernatant of lipopolysaccharide (LPS)-stimulated human PBMC-differentiated macrophages**. Human PBMC-differentiated macrophages were pre-incubated with 5 µg/ml hS9-Fab03 for 2 h, then stimulated with 1 µg/ml LPS. Anthrax chimeric Fab antibody was used as the negative control. The production of IFN-β **(A)**, TNF-α **(B)**, IL-1β **(C)**, IL-6 **(D)**, IL-8 **(E)**, and IL-10 **(F)** in human PBMC-differentiated macrophage culture supernatant were determined by ELISA analysis. Data are shown as mean ± SD (*n* = 3, N.S., not significant, *p < 0.05, **p < 0.01, ***p < 0.001 compared to negative control).

Next, we investigated whether hS9-Fab03 ligation could regulate the expression of pro-inflammatory cytokines in human macrophages. The THP-1-differentiated macrophages were pretreated with the hS9-Fab03 and stimulated with LPS for different time. The mRNAs were collected to evaluate the expression of pro-inflammatory cytokines. We found that hS9-Fab03 could obviously inhibit LPS-initiated production of pro-inflammatory cytokines, such as TNF-α, IL-6, and IFN-β, no effect on the expression of IL-1β, IL-8, and IL-10 in THP-1-differentiated macrophages (Figures 3 and 4). Similarly, we observed that pre-incubation of hS9-Fab03 could also suppress the TNF-α, IL-6, IL-1β, IL-8, and IFN-β expression of pro-inflammatory in human PBMC-derived macrophages stimulated with LPS, but slightly increased IL-10 production in an early time point compared to group with LPS only (Figures 5 and 6). We also incubated different concentrations of hS9-Fab03 macrophages and found that hS9-Fab03 could effectively block LPS-induced TNF-α and IFN-β expression in a dose-dependent manner (Figures S1A,B in Supplementary Material). Thus, the results implied that the hS9-Fab03 could inhibit LPS-induced pro-inflammatory cytokines production in human macrophages.

### Suppression of LPS-Initiated TLR4 Signaling by the hS9-Fab03

**Figure 7:**
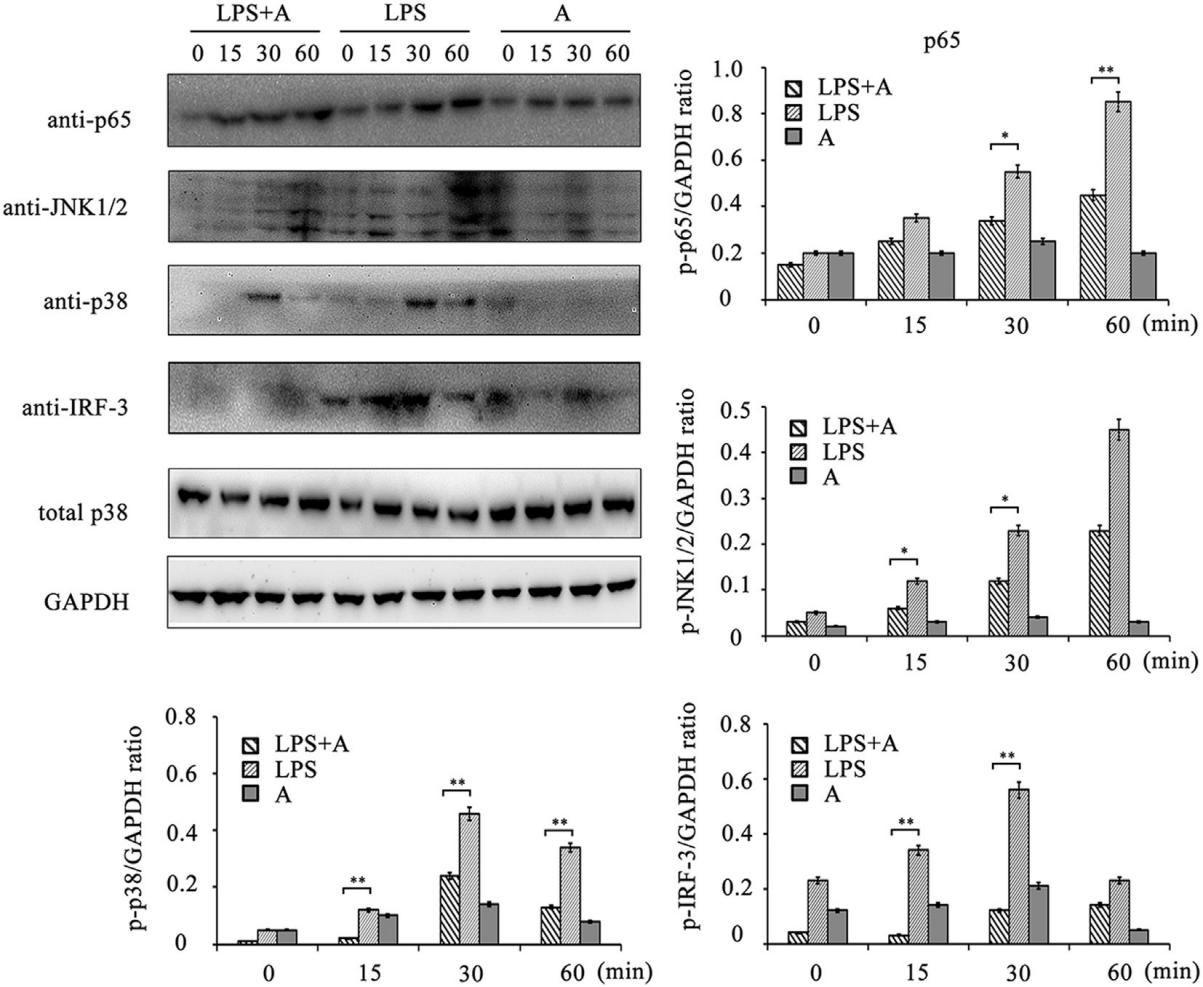
**Western blot analysis for inhibition of lipopolysaccharide (LPS)-induced NF-κB, MAPKs, and IRF-3 activation by hS9-Fab03**. Cells were *pretreated* with 5 µg/ml of the hS9-Fab03 for 2 h and further incubated in presence or absence of LPS (1 µg/ml). After immunoblotting, the phosphorylation levels of p65, JNK1/2, p38, and IRF-3 were identified using phosphor-specific antibodies. Total p38 and GAPDH were used to ensure equal loading. L, LPS; A, hS9-Fab03. Data are shown as mean ± SD (*n* = 3, N.S., not significant, *p < 0.05, **p < 0.01, ***p < 0.001 compared to negative control).

To investigate the inhibitory effects of the hS9-Fab03 on TLR4 signaling transduction induced by LPS ligation, we detected the phosphorylation levels of NF-κB, MAPKs, and IRF-3 signaling pathways. As shown in Figure 7, LPS ligation significantly increased the phosphorylation level of these proteins in THP-1-differentiated macrophages, but the phosphorylation level of p65, p38, JNK1/2, and IRF-3 was suppressed when pretreated with 5 µg/ml of the hS9-Fab03. Thus, these results demonstrated that the hS9-Fab03 could suppress LPS-triggered TLR4 signaling transduction.

## Discussion

In this study, we utilized the human Fab phage library to construct an active anti-Siglec-9 antibody Fab fragments (hS9-Fab03) that could recognize recombinant Siglec-9 protein. The screening strategy was applied by the repeated panning with coated recombinant Siglec-9 protein in microtiter plates to assure the efficiency of specific Siglec-9 binding phage. After seven rounds of screening, 10 of 50 selected candidate phage clones displayed strong positive signal, and ELISA results indicated that these clones possess the specifically binding ability. After this, the prokaryotic expression vector pETDuet-hSiglec-9 Fab03 was successfully constructed. Our reliable approach ensured that the two different chains are comparatively expressed and efficiently dimerized into active hS9-Fab03, which ensured the production of stable antibody fragments (32). Series of western blotting and SDS-PAGE with Coomassie brilliant blue staining confirmed the efficient expression and purification of hS9-Fab03. Moreover, FACS confirmed the specifically binding ability of hS9-Fab03 to Siglec-9 protein expressed on the surface of THP-1-differentiated macrophages. Thus, these results demonstrated that the antibody engineering process couldn’t change the specificity of the human anti-Siglec-9 Fab fragments.

Sepsis is a systemic infection characterized by the release of pro-inflammatory cytokines inducing systemic immune activation and coagulation activation and causing severe sepsis and septic shock (33). TLRs are fundamental for the initiation of the protective pro-inflammatory responses, which served as the first line of host defense against infection (34, 35). The uncontrolled pro-inflammatory cytokine production is critically involved in a variety of pathologies, such as sepsis and autoimmune diseases (36). TLR4 were mainly responded to PAMPs (such as LPS) and DAMPs (such as HMGB1), whose excessive activation could stimulate macrophages, DCs, and neutrophils which play a vital role in adaptive immunity by contributing to sepsis (37, 38). Accumulating data showed that inhibition of TLR4 signaling by means of antibodies or soluble decoy receptors have proven to obtain a clinical benefit to sepsis (39). In this study, we found that the production of TNF-α, IL-1β, IL-6, IL-8, and IFN-β were downregulated in hS9-Fab03-treated LPS-stimulated human macrophages compared with control group, but slightly increased IL-10 production in an early time point. The downregulation of pro-inflammatory cytokines and the enhanced expression of IL-10 could be beneficial in preventing macrophage activation. It is well established that C/EBP and Sp1 are involved in activating the expression of IL-10 after stimulation by LPS (40). As shown in Figure 5F, pretreatment of hS9-Fab03 could enhance the IL-10 expression after the LPS stimulation, which might contribute to the late tolerance in human macrophages. Siglecs could negatively regulate innate immunity by recruiting tyrosine phosphatase SHP-1 and -2 through their ITIMs (41). However, we did not detect the phosphorylation level of SHP-1 or -2 in the present study. Thus, the exact roles of hS9-Fab03 in enhanced production of IL-10 remain to be further investigated.

Human antibodies are broadly used in clinical diagnosis and treatment of diseases, which are produced by various DNA recombination technologies. Meanwhile, the phage display utilizes the advantage of being inexpensive and efficient. Previously, we have constructed a fully human Fab phage library for Fab investigation, then smaller antibody fragments (Fabs or scFvs) could be comprised with phage display (9, 28). Considerable results have showed that these fragments preserve high efficiency in penetrating into the targeted tissue with high concentration and validity (42).

The exact mechanism of how hS9-Fab03 block TLR4 signaling transduction is still unknown. One possibility is that hS9-Fab03 could directly bind to the Siglec-9 and induce the activation of SHP-1 or SHP-2, whose tyrosine phosphatase activity might inhibit the phosphorylation of downstream molecules of TLR4 signaling pathway. In this case, the inhibition of LPS-induced IFN-β and TNF-α levels were increased in an antibody concentration-dependent manner (Figures S1A,B in Supplementary Material), while Western blotting data showed that hS9-Fab03 could suppress the phosphorylation of key molecules downstream of TLR4 pathway as shown in Figure 7. Additional studies should be performed to clarify the mechanism and exploit its biological effects *in vivo*.

In conclusion, we introduce the preparation and characteristics of a human anti-Siglec-9 antibody Fab fragment (hS9-Fab03), in this study, which could specifically bind to Siglec-9 with high affinity. Pretreatment with hS9-Fab03 could suppress LPS-stimulated TLR4 signaling transduction and inhibit the production of pro-inflammatory cytokines in human macrophages. Our data suggest ligation of Siglec-9 with hS9-Fab03 might be a novel anti-inflammatory therapeutic strategy for sepsis.

## Author Contributions

MW, JZ, and XZ designed the experiments. SC, XZ, NY, WZ, BC, YW, and QS performed the experiments. SC, XZ, NY, WZ, FZ, and SN analyzed the data. ZY, XZ, and CW contributed reagents/materials/analysis tools. SC and XZ wrote the paper.

## Conflict of Interest Statement

The authors declare that the research was conducted in the absence of any commercial or financial relationships that could be construed as a potential conflict of interest.

## Supplementary Material

Figure S1 **The optimization of pre-incubation time and concentration of hS9-Fab03 and lipopolysaccharide (LPS)**. **(A,B)** PMA-stimulated THP-1-differentiated macrophages in 24 wells were pre-incubated with different concentrations of hS9-Fab03 for 2 h and then were stimulated with 1 µg/ml LPS. **(C,D)** The macrophages in 24 wells were pre-incubated with 5 µg/ml hS9-Fab03 with different times then stimulated with 1 µg/ml LPS. **(E,F)** The macrophages in 24 wells were pre-incubated with 5 µg/ml hS9-Fab03 for 2 h and then stimulated with different concentrations of LPS. The mRNA expression levels of IFN-β and TNF-α were detected by Q-PCR. Anthrax chimeric Fab antibody was used as the negative control. Data are shown as mean ± SD (*n* = 3, N.S., not significant, **p* < 0.05, ***p* < 0.01, ****p* < 0.001 compared to negative control).

Table S1 **Primers used for the construction of the hS9-Fab03 gene**.
