# Supplementary figures and images for: The Fab Fragment of a Human Anti-Siglec-9 Monoclonal Antibody Suppresses LPS-Induced Inflammatory Responses in Human Macrophages
Source: Front Immunol. 2016 Dec 26;7:649. doi: 10.3389/fimmu.2016.00649 (PMC5183739; doi:10.3389/fimmu.2016.00649)

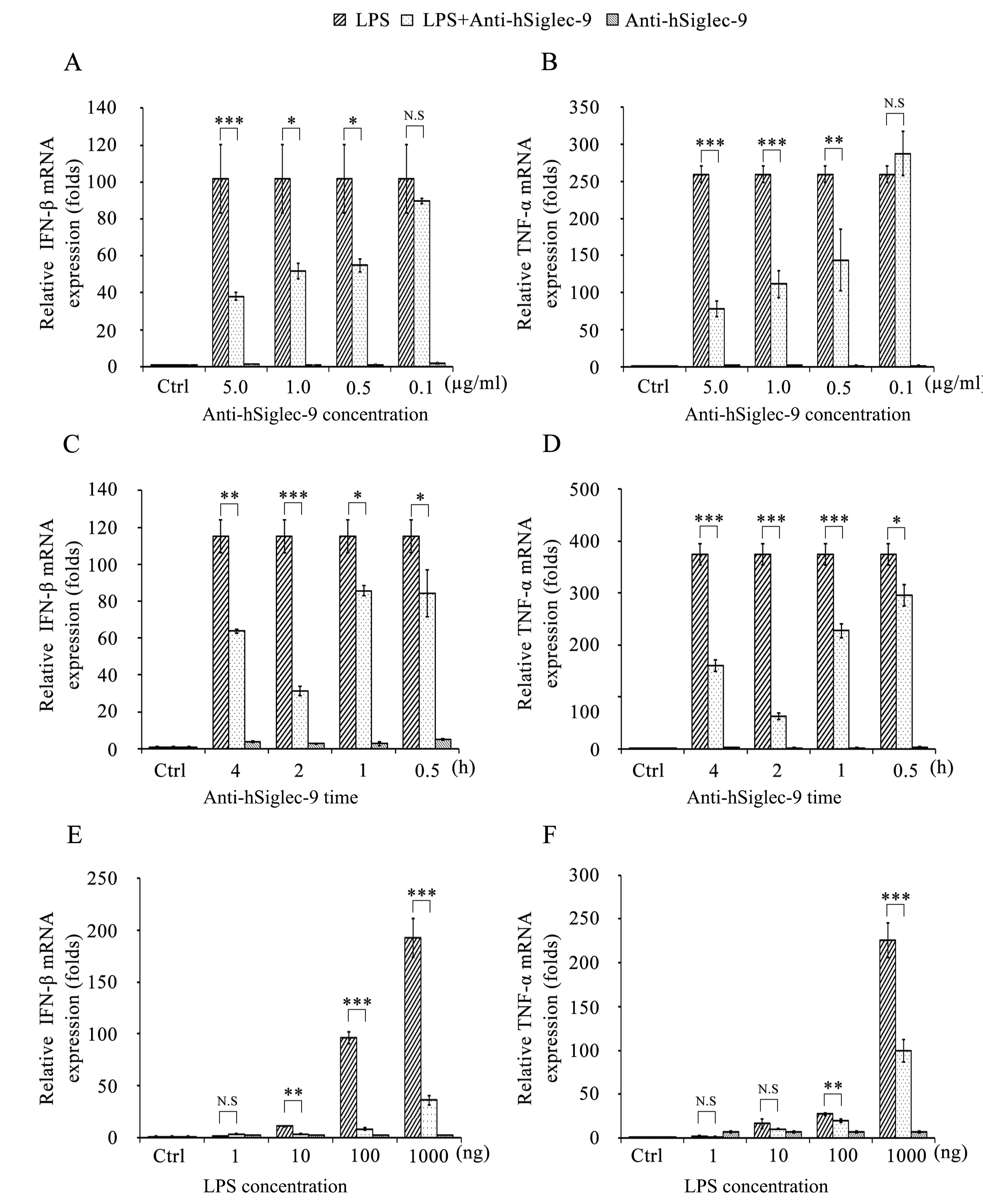

Supplement: Figure S1 — The optimization of pre-incubation time and concentration of hS9-Fab03 and lipopolysaccharide (LPS). (A,B) PMA-stimulated THP-1-differentiated macrophages in 24 wells were pre-incubated with different concentrations of hS9-Fab03 for 2 h and then were stimulated with 1 µg/ml LPS. (C,D) The macrophages in 24 wells were pre-incubated with 5 µg/ml hS9-Fab03 with different times then stimulated with 1 µg/ml LPS. (E,F) The macrophages in 24 wells were pre-incubated with 5 µg/ml hS9-Fab03 for 2 h and then stimulated with different concentrations of LPS. The mRNA expression levels of IFN-β and TNF-α were detected by Q-PCR. Anthrax chimeric Fab antibody was used as the negative control. Data are shown as mean ± SD (n = 3, N.S., not significant, *p < 0.05, **p < 0.01, ***p < 0.001 compared to negative control). [file image_1.tif]
