# Supplementary material for: The Fab Fragment of a Human Anti-Siglec-9 Monoclonal Antibody Suppresses LPS-Induced Inflammatory Responses in Human Macrophages
Source: Front Immunol. 2016 Dec 26;7:649. doi: 10.3389/fimmu.2016.00649 (PMC5183739; doi:10.3389/fimmu.2016.00649)
Supplement: Table S1 — Primers used for the construction of the hS9-Fab03 gene. [file table_1.doc]

Table1. Primers used for the construction of the hS9-Fab03 gene.

| **Primer nameDNA sequence** | |
| --- | --- |
| Heavy chain variable region forward primer | |
| VH1 | GCTGCCCAACCAGCCATGGCCCAGGTGCAGCTGGTGCAGTCTGG |
| VH2 | GCTGCCCAACCAGCCATGGCCCAGATCACCTTGAAGGAGTCTGG |
| VH3 | GCTGCCCAACCAGCCATGGCCGAGGTGCAGCTGGTGSAGTCTGG |
| VH4 | GCTGCCCAACCAGCCATGGCCGAGGTGCAGCTGKTGGAGTCTG |
| VH5 | GCTGCCCAACCAGCCATGGCCCAGGTGCAGCTGVAGGAGTCGGG |
| VH6 | GCTGCCCAACCAGCCATGGCCCAGGTGCAGCTACAGCAGTGGGG |
| Heavy chain variable region reverse primer | |
| VH7 | CGATGGGCCCTTGGTGGAGGCTGAGGAGACGGTGACCAGGGTTCC |
| VH8 | CGATGGGCCCTTGGTGGAGGCWGRGGAGACGGTGACCAGGGTBCC |
| Light chain variable region forward primer | |
| VL1 | GGGCCCAGGCGGCCGAGCTCGTGBTGACACAGCCGCCCTC |
| VL2 | GGGCCCAGGCGGCCGAGCTCGTGCTGACTCAGCCACCCTC |
| VL3 | GGGCCCAGGCGGCCGAGCTCGCCCTGACTCAGCCTCCCTCCGT |
| VL4 | GGGCCCAGGCGGCCGAGCTCGAGCTGACTCAGCCACCCTCAGTGT |
| VL5 | GGGCCCAGGCGGCCGAGCTCGTGCTGACTCAATCGCCCTC |
| VL6 | GGGCCCAGGCGGCCGAGCTCATGCTGACTCAGCCCCACTC |
| VL7 | GGGCCCAGGCGGCCGAGCTCGTGGTGACYCAGGAGCCMTC |
| VL8 | GGGCCCAGGCGGCCGAGCTCGTGCTGACTCAGCCACCTTC |
| VL9 | GGGCCCAGGCGGCCGAGCTCGGGCAGAACTCAGCAGCTCTC |
| Light chain variable region reverse primer | |
| VL10 | CGAGGGGGCAGCCTTGGGCTGACC |
| Heavy chain variable region primer | |
| HF1 | CCGGATATCGCAGGTGCAGCTGGTGCAGTCTGG |
| HR1 | TGGGCCCTTGGTGGAGGCTGAGGAGACGGTGACCAGGG |
| Light chain variable region primer | |
| LL1 | CATGCCATGGGCCAGTCTGCCCTGACTCAGCCCC |
| LR1 | CCTCAGAGGAGGGCGGGAACAGAGTGACCGAGGGGGCAGCCTTG |
| Constant region CH primer | |
| HF2 | CCCTGGTCACCGTCTCCTCAGCCTCCACCAAGGGCCCA |
| HR2 | CCGCTCGAGTTAAGAAGCGTAGTCCGGAACGTCG |
| Constant region CL primer | |
| LL2 | CCAAGGCTGCCCCCTCGGTCACTCTGTTCCCGCCCTCCTCTGAGG |
| LR2 | CCCAAGCTTTTATGAACATTCTGTAGGGGCCAC |
| Heavy chain primer | |
| HF1 | CCGGATATCGCAGGTGCAGCTGGTGCAGTCTGG |
| HR2 | CCGCTCGAGTTAAGAAGCGTAGTCCGGAACGTCG |
| Light chain primer | |
| LL1 | CATGCCATGGGCCAGTCTGCCCTGACTCAGCCCC |
| LR2 | CCCAAGCTTTTATGAACATTCTGTAGGGGCCAC |
